# Erratum to “Reproductive performance of early- and late-calving dairy cows artificially inseminated after ovulation synchronization and estrous resynchronization or artificially inseminated after observed estrus” (JDS Commun. 2:80–85)

**DOI:** 10.3168/jdsc.2022-3-2-165

**Published:** 2022-03-16

**Authors:** S.G. Moore, S.A. Hamilton, R. Molina-Coto, L.M. Mayo, R.O. Rodrigues, T. Leiva, S.E. Poock, M.C. Lucy

The animal ethics statement was missing from this paper. The following statement should be included: “The project work was reviewed for ethical treatment of animals and approved by the Animal Care and Use Committee (ACUC) of the University of Missouri.”

## References

[bib1] Moore S.G., Hamilton S.A., Molina-Coto R., Mayo L.M., Rodrigues R.O., Leiva T., Poock S.E., Lucy M.C. (2021). Reproductive performance of early- and late-calving dairy cows artificially inseminated after ovulation synchronization and estrous resynchronization or artificially inseminated after observed estrus. JDS Commun..

